# Targeted Dereplication of *H. patulum* and *H. hookeranium* Extracts: Establishing MS/MS Fingerprints for the Identification of Polycyclic Polyprenylated Acylphloroglucinols

**DOI:** 10.3390/molecules30122531

**Published:** 2025-06-10

**Authors:** Annabelle Dugay, Florence Souquet, David Hozain, Gilles Alex Pakora, Didier Buisson, Séverine Amand, Marie-Christine Lallemand, Raimundo Gonçalves de Oliveira Junior

**Affiliations:** 1 Cibles Thérapeutiques et Conception de Médicaments (CiTCoM UMR CNRS 8038), Faculty of Pharmacie, Université Paris Cité, 75006 Paris, France; annabelle.dugay@u-paris.fr (A.D.); florence.souquet@u-paris.fr (F.S.); hozaind@gmail.com (D.H.); gilles.pakora@u-paris.fr (G.A.P.); marie-christine.lallemand@u-paris.fr (M.-C.L.); 2 Molécules de Communication et Adaptation des Micro-Organismes (MCAM UMR CNRS 7245), Muséum National d’Histoire Naturelle (MNHN), 75005 Paris, France; didier.buisson@mnhn.fr (D.B.); severine.amand@mnhn.fr (S.A.)

**Keywords:** dereplication, *Hypericum*, metabolomics, phloroglucinol derivatives, phenolic compounds

## Abstract

In this study, we combined automated annotation tools with targeted dereplication based on MS/MS fragmentation pathway studies to identify polycyclic polyprenylated acylphloroglucinols (PPAPs) in *Hypericum* species, using *H. patulum* and *H. hookeranium* as a case study. These species, extensively used in traditional medicine, exhibit morphological similarities that often result in misidentification. Following UHPLC-HRMS/MS analysis of plant extracts, a molecular network approach facilitated a comprehensive comparison of their chemical composition, assigning specific clusters to *O*-glycosylated flavonoids and PPAPs. Eight peaks, including quercitrin, isoquercitrin, procyanidins, chlorogenic acid, quercetin, and glycosylated derivatives, were annotated from the GNPS database. For PPAPs, despite the structural complexity posing challenges for automated annotation using public databases, our targeted-dereplication strategy, relying on in-house spectral data, led to the putative identification of 22 peaks for *H. patulum* and *H. hookeranium*. Key compounds such as hyperforin, hyperscabrone K, and garcinialliptone M were detected in both species, underscoring their chemical similarity. MS/MS fragmentation pathways, particularly the successive losses of isobutene and isoprenyl units, emerged as a consistent signature for PPAP detection and may be useful for selecting PPAP-enriched extracts or fractions for further phytochemical investigations.

## 1. Introduction

*Hypericum* is a genus of flowering plants from the family Hypericaceae, comprising about 400 species, which are mostly perennial herbs or shrubs. These plants are known for their yellow flowers and have a long history of use in traditional medicine for various therapeutic purposes [1,2]. Indeed, many *Hypericum* species have shown a broad range of pharmacological activities, such as anti-inflammatory, anxiolytic, antidepressant, antiviral, and antibacterial effects [3,4,5,6]. Previous phytochemical investigations have resulted in the discovery of xanthones, flavonoids, volatile compounds, phloroglucinol derivatives, and other components [7,8,9,10].

Polycyclic polyprenylated acylphloroglucinols (PPAPs) constitute a unique class of metabolites common to this genus and have become a research hotspot in the field of natural product chemistry due to their complex structures. PPAPs can be divided into bridged-cyclic with bicyclo[3.3.1]nonane-2,4,9-trione cores, adamantane, homoadamantane, spirocyclic, and some other complicated architectures based on their different scaffolds [11]. This molecular diversity justifies their various biological effects and highlights them as major contributors to the therapeutic purposes of *Hypericum*.

Although the isolation of new PPAPs has been extensively reported, the use of analytical tools that enable the rapid identification of known molecules, guiding the search for novel ones, remains a significant challenge. This is because many PPAPs exhibit diverse substitution patterns. They vary in the number of isoprenyl/geranyl units carried by the bicyclic/phloroglucinol core, the presence of catechol groups, rearrangements, and basic skeleton or side chains oxidation [11]. The use of more assertive techniques, e.g., nuclear magnetic resonance (NMR), is essential for unambiguous identification and is strongly recommended for the structural determination of purified PPAPs. However, the identification of PPAPs in mixtures via NMR can be truly problematic, given the technique’s low sensitivity to minor compounds, as well as the cost and time required for analyses with higher resolution.

Recent advances in liquid chromatography coupled with high-resolution tandem mass spectrometry (HRMS^n^) have significantly facilitated the detection of several known compounds simultaneously in an untargeted manner, even if they are present in low quantity. The use of public platforms for automated MS/MS annotation, such as GNPS (Global Natural Product Social Molecular Networking), has tremendously contributed to the identification of organic compounds in matrices of all kinds, with a high level of confidence [12]. Combined with molecular networks, these platforms are capable of reorganizing spectral data, establishing chemical fingerprints, and helping to understand the influence of biotic and abiotic factors on the plant metabolome and how this interferes with its therapeutic applications [13,14]. Nevertheless, the arsenal of compounds recorded in these platforms is still limited and does not encompass all groups of metabolites, as the task of populating the database relies on the voluntary work of its users. Experimental data from different equipment can also result in highly distinct fragmentation profiles for the same molecule. Non-trivial compounds, occurring in limited genera or even species, often remain unannotated. Consequently, establishing fragmentation profiles for these molecules becomes indispensable in identifying compounds that may not be automatically recognized.

Considering the structural complexity of PPAPs and the limited availability of spectral data in public databases, this study presents an approach that integrates automated annotation tools with targeted dereplication based on MS/MS fragmentation pathway analysis. Targeted dereplication involves the guided identification of specific known compounds within complex mixtures, using prior knowledge such as diagnostic fragmentation patterns. In this context, we introduce a strategy specifically tailored to PPAPs. Using hyperforin—a well-known PPAP—as a model compound, we propose a fragmentation-guided annotation workflow grounded in consistent MS/MS fragmentation patterns. This methodological contribution addresses a critical gap in current dereplication practices and provides a foundation for expanding PPAP representation in public spectral libraries. To demonstrate the approach, we selected *H. patulum* and *H. hookeranium* as model species, both widely used in traditional medicine and frequently misidentified due to their morphological similarities. The establishment of MS/MS fingerprints for these species offers a practical and rapid tool for PPAP detection and selection of PPAP-rich samples for targeted isolation and detailed structural characterization.

## 2. Results and Discussion

**Figure 1:**
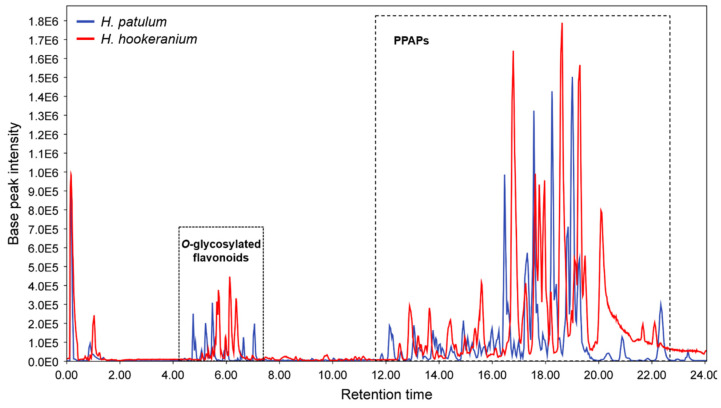
Base Peak Chromatogram (BPC) of *H. patulum* (blue) and *H. hookeranium* (red) methanol extracts using UHPLC-HRMS/MS in positive ionization mode (ESI+).

The objective of this study was to combine automated annotation tools with targeted dereplication based on MS/MS fragmentation pathway studies to identify PPAPs in different *Hypericum* species. Initially, a UHPLC-HRMS/MS analysis method was performed to separate the main classes of secondary metabolites found in *Hypericum*, particularly *O*-glycosylated flavonoids and PPAPs. As shown in Figure 1, the Base Peak Chromatogram (BPC) obtained with MS detection provided the first molecular fingerprint of the extracts, highlighting their complexity and differentiating both chemical groups based on their polarity. Positive ionization mode with an ESI source was chosen because it allowed the ionization of more compounds than the negative one and a good fragmentation of the molecular ions from both flavonoids and PPAP groups, giving suitable MS/MS spectra.

**Figure 2:**
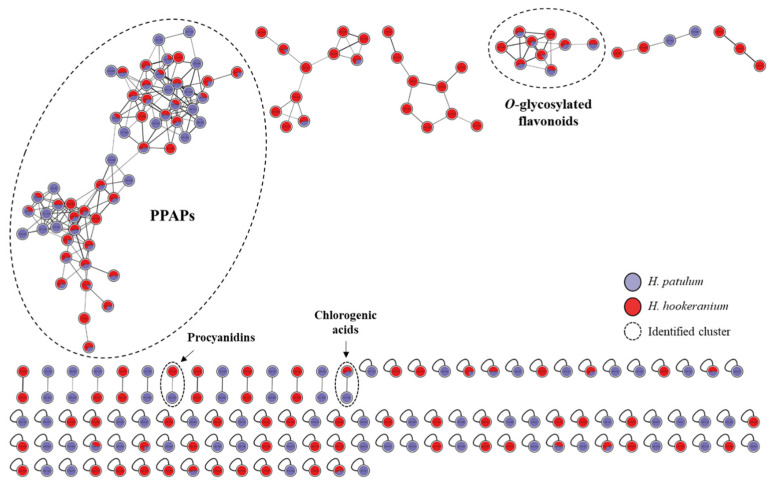
Molecular network of *H. patulum* (purple) and *H. hookeranium* (red) extracts using GNPS platform and visualized with Cytoscape 3.10.1 software. A high-resolution version of the figure is in the supporting information (Figure S6).

The differentiation of these two predominant classes of metabolites in both extracts is also observed in the molecular network presented in Figure 2. Indeed, the organization of spectral data into molecular networks enables a more comprehensive comparison of the chemical composition of the two investigated species and the attribution of clusters to specific groups of compounds and provides information about the relative distribution of the same metabolite in various samples. This is because molecules exhibiting the same fragmentation pattern (identical fragments and/or similar mass losses) are grouped in the same cluster, whereas substances with different MS/MS spectra are not associated [13].

**Figure 3:**
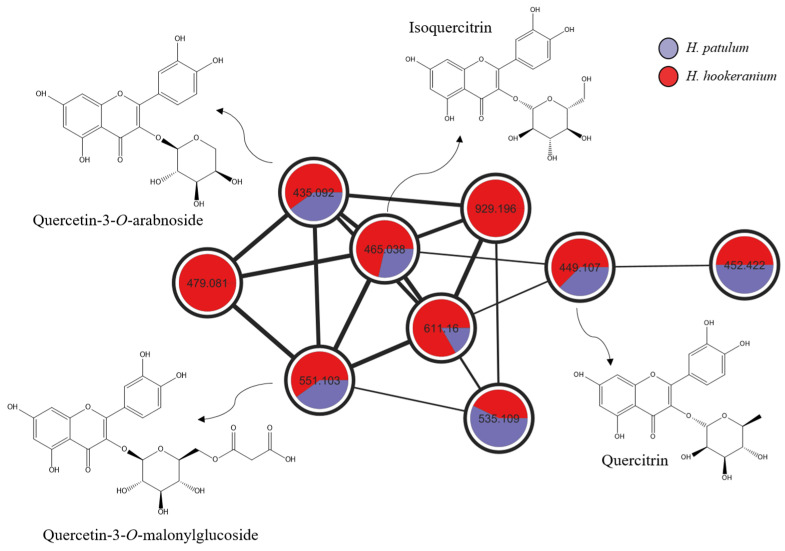
Cluster of O-glycosylated flavonoids annotated according to spectral similarity indicated by GNPS and confirmed by the literature data.

As anticipated, the molecular network obtained revealed the presence of two main clusters common to both investigated species: *O*-glycosylated flavonoids and PPAPs. Small clusters containing only a few nodes were not identified. After comparison with spectral data from the GNPS library and the literature, it was possible to annotate eight peaks, including four *O*-glycosylated flavonoids, quercetin, procyanidins B1 and B2, and chlorogenic acid (Table 1). The *O*-glycosylated flavonoids cluster is presented in Figure 3 and shows the annotation of quercitrin, isoquercitrin, quercetin-3-*O*-malonylglucoside, and quercetin-3-*O*-arabnoside, all 5,7,3′,4′-tetrahydroxy-3-*O*-glycoslytated flavones sharing a 303.05 *m*/*z* fragment attributed to the aglycone (quercetin). The other phenolic compounds annotated based on spectral similarity, indicated by GNPS (cosine score > 0.75) and compared to the literature data, are commonly found in *Hypericum* species, as previously reported [15,16].

Calculated molecular formulas for the detected parent ions and mass losses assigned to isoprenyl units allowed attributing the most significant cluster in the molecular network to PPAPs. Contrary to phenolic compounds, a comparison with GNPS spectral data did not enable the automatic annotation of any nodes from the PPAP cluster, probably due to their structural complexity and/or the absence of MS/MS data in the database. Therefore, dereplication was carried out based on the spectral data from the in-house database by establishing a fragmentation study for each proposed structure. As summarized in Table 2 and Table 3, twenty-two peaks were annotated for *H. patulum* (fourteen compounds) and *H. hookeranium* (eight compounds). Some of them were detected in both plants (e.g., hyperforin/hypercohin G, hyperscabrone K, garcinialliptone M) and are commonly found in *Hypericum* [7,11]. Note that some compounds were identified as isomers when represented by peaks with similar precursor ions and MS/MS spectra but with significantly different retention times.

**Figure 4:**
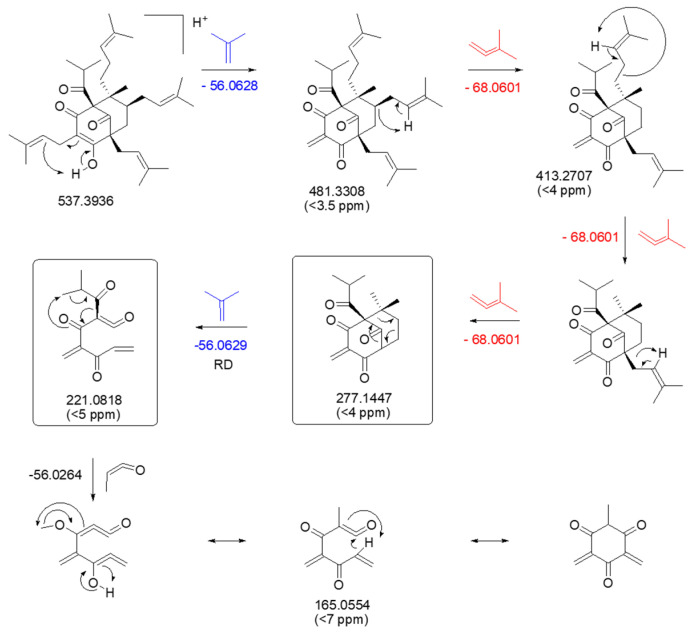
MS/MS-ESI spectrum in positive mode ionization and fragmentation pathway proposed for hyperforin detected in both *H. patulum* and *H. hookeranium* extracts.

The starting point of the following fragmentation study is hyperforin, an acyl-isopropyl-substituted PPAP common to both species and considered a chemotaxonomic marker of the genus. We compared the spectral data of the peak assigned to hyperforin with those obtained for a previously isolated and fully characterized standard compound [21] (NMR spectra are provided in the supporting information, Figure S7). As shown in Figure 4, the fragmentation of the [M + H]^+^ molecular ion at *m*/*z* 537.3937 (C_35_H_52_O_4_) triggers the loss of an isobutene moiety (C_4_H_8_, *m*/*z* 56.0636) after McLafferty rearrangement, resulting in the ion *m*/*z* 481.3301. Two isoprenyl units (*m*/*z* 68.0624) are subsequently lost through the same mechanism, leading to the formation of *m*/*z* 413.2677 and 345.2053, respectively. From this last ion, two fragmentation pathways emerge. In the first one, *m*/*z* 277.1426 (base peak) is obtained after the loss of the third isoprenyl group (*m*/*z* 68.0624). A retro Diels-Alder (RD) rearrangement explains the loss of another C_4_H_8_ unit (*m*/*z* 56.0630), resulting in the ion *m*/*z* 221.0803. The loss of a C_3_H_4_O unit (*m*/*z* 56.0260) provides the final fragment at *m*/*z* 165.0541. The second pathway displayed fragments attributed to consecutive losses of H_2_O (*m*/*z* 259.1323) and C_4_H_8_ (*m*/*z* 203.0698).

**Figure 5:**
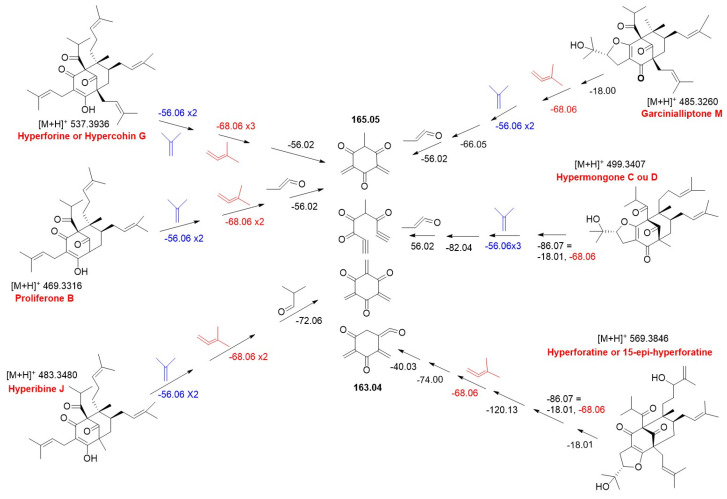
Common MS/MS fragmentation (*m*/*z*) pathway proposed to acyl-isopropyl-substituted PPAPs putatively identified in *Hypericum* extracts.

From the hyperforin spectral data, other acyl-isopropyl-substituted PPAPs were putatively identified, and a single MS/MS fragmentation scheme was proposed (Figure 5). Successive losses of isobutene and isoprenyl (−56.06 and −68.06, respectively) moieties are found in all fragmentation pathways of the PPAPs proposed in this study, constituting a true MS/MS signature for their detection. Note that the fragmentation schemes for the [M + H]^+^ molecular ions at *m*/*z* 469.3308 (C_30_H_44_O_4_) and 483.3480 (C_31_H_46_O_4_) detected in *H. patulum* are similar to those of hyperforin (Figure 5). They differ essentially in the number of isoprenyl units anchored to the core of the molecule or in their substitution by some other alkyl group. Understanding this fragmentation pattern, combined with information from the literature, these peaks were annotated as prolifenone B [22] and hyperibine J [23], respectively. Garcianialliptone M and hypermongone C or D, as well as hyperforatin or 15-epi-hyperforatin, were also putatively identified in this group. All acyl-isopropyl-substituted PPAPs share the same basic core, represented by a final fragment at *m*/*z* 165.05 or its rearranged equivalent at *m*/*z* 163.04.

**Figure 6:**
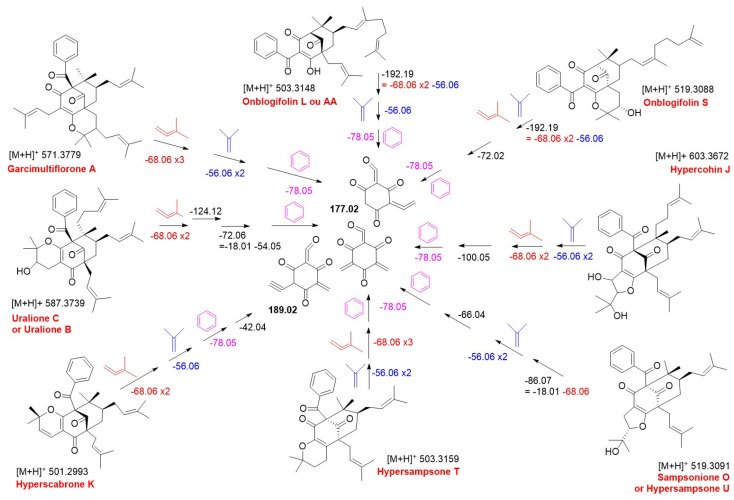
Common MS/MS fragmentation (*m*/*z*) pathway proposed to acyl-phenyl-substituted PPAPs putatively identified in *Hypericum* extracts.

MS/MS spectra of the [M + H]^+^ molecular ions at *m*/*z* 603.3672 (C_38_H_50_O_6_), 587.3739 (C_38_H_50_O_5_), 519.3091 (C_33_H_42_O_5_), 503.3149 (C_33_H_42_O_4_), and 571.3779 (C_38_H_50_O_4_), predominantly detected in *H. patulum*, exhibited common final fragments (*m*/*z* 255.06 and 177.02), suggesting that they have a similar fragmentation pattern. Besides the successive losses of isobutene and isoprenyl units, a loss of a phenyl moiety (*m*/*z* 78.05) was observed in all compounds of the group, contributing to their annotation and placement within the PPAP cluster. The proposed structures were compatible with hypercohin J (*m*/*z* 603.3672), uralione B or C (*m*/*z* 587.3739), sampsonione O or hypersampsone U (*m*/*z* 519.3088), hypersampsone T (*m*/*z* 503.3148), and garcimultiflorone A (*m*/*z* 571.3779). Figure 6 shows a common fragmentation pathway, illustrating the detection of key fragments and mass loss for the acyl-phenyl-substituted PPAPs.

**Figure 7:**
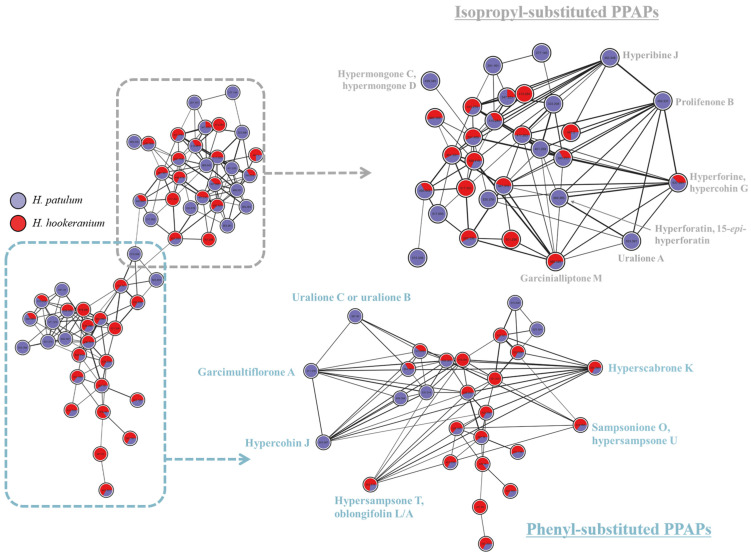
Cluster of PPAPs annotated according to spectral similarity to the literature data. Two sub-clusters were distinguished: (1) acyl-isopropyl-substituted derivatives (grey) and (2) acyl-phenyl-substituted derivatives. Some nodes were dragged outward for better visualization.

When isolated, it becomes evident that the PPAP cluster presented in Figure 7 harbors two distinct “sub-clusters”, attributed to the described acyl-isopropyl and acyl-phenyl-substituted derivatives. The grouping of nodes carrying the same substituent (acyl or phenyl) supports the proposed annotation for these compounds and the mechanistic hypothesis given in their respective fragmentation pathways.

Other compounds exhibiting alternative fragmentation pathways have also been identified. After spectral data analysis, a molecular ion [M + H]^+^ at *m*/*z* 567.4050 (C_36_H_54_O_5_) was assigned to hyphenrone E. The presence of different alkyl groups linked to the phloroglucinol core and the high degree of oxygenation and cyclization of the prenyl chains contributed to an atypical fragmentation pattern compared to the two groups of PPAPs described earlier. Nevertheless, successive losses of isobutene and isoprenyl units are consistently observed, confirming that they are the main spectral signature for the detection of PPAPs by mass spectrometry (supporting information).

The detection of PPAPs common to both species indicates that the similarities between them are not only morphological but also chemical, justifying the frequent confusion in their identification. However, some compounds annotated only for *H. patulum* (uralione A, hypercohin J, uralione B or C, hyphenrone E, hypersampsone T, hyperforatin or 15-*epi*-hyperforatin, hypermongone C or D, prolifenone B, and hyperibine J) or *H. hookeranium* (oblongifolin S and oblongifolin L or A) may aid in their distinction. For instance, the uraliones detected exclusively in *H. patulum* are commonly isolated from *H. uralum*, which is considered a botanical synonym of *H. patulum* var. *uralum* [24]. This taxonomic relationship may explain the exclusive presence of such compounds in the *H. patulum* extract, reflecting not only morphological but also chemical similarities between these closely related taxa. On the other hand, oblongifolin-type compounds found only in *H. hookeranium* are typically reported in species of the genus *Garcinia* [25,26]. This suggests a potentially unique biosynthetic capacity in *H. hookeranium*, a species that remains comparatively understudied in terms of its phytochemical profile when compared to other *Hypericum* species. Nevertheless, the chemical differentiation between taxa based solely on shared or exclusive PPAPs should be interpreted with caution. Robust confirmation of these preliminary observations would require chemometric analyses and a broader metabolomic dataset to support statistically validated comparisons.

Despite the LC-MS/MS dereplication approach addressed in this study being useful for PPAP identification, we emphasize that classical phytochemical investigations involving the purification and complete structural elucidation using more sensitive techniques (such as NMR) remain indispensable for unequivocal characterization. In this sense, the MS/MS fingerprints highlighted in this study can be valuable in selecting extracts or fractions rich in PPAPs for further phytochemical studies.

From a pharmacological point of view, several of the putatively identified PPAPs in this study have been reported to exhibit significant activities. For instance, hyperforin, a well-known acyl-isopropyl PPAP, has demonstrated notable antidepressant, anti-inflammatory, and antimicrobial properties, largely attributed to its capacity to modulate neurotransmitter uptake and inflammatory mediators [27,28,29]. The presence of other PPAPs, such as hyphenrone E, and phenolic compounds, such as quercitrin, isoquercitrin, and chlorogenic acid, has been associated with the cytotoxic and antiplasmodial activities of several *Hypericum* species, including *H. sampsonii* [30,31]. These bioactivities reflect the rich pharmacological potential of *Hypericum*-derived PPAPs and justify the continued exploration of these compounds. Thus, the MS/MS-based dereplication and fingerprinting approach employed here not only facilitates the rapid identification of structurally complex metabolites but also supports future studies aiming to isolate and evaluate the therapeutic relevance of these molecules.

## 3. Materials and Methods

### 3.1. Chemicals and General Procedures

HPLC PLUS grade methanol and LC-MS grade acetonitrile were purchased from Carlo Erba Ltd. (Milan, Italy). LC-MS grade 99% formic acid was purchased from SERVA Electrophoresis GmbH (Heidelberg, Germany).

### 3.2. Plant Material

Fresh leaves of *H. patulum* were obtained from the botanical collection at the *Muséum National d’Histoire Naturelle* (MNHN, Paris, France), whereas leaves of *H. hookeranium* were collected from a private garden and identified by botanists at the MNHN through comparison with a voucher specimen (#124870).

### 3.3. Plant Extraction

Immediately after harvesting, the fresh leaves were pulverized with liquid nitrogen. HPLC PLUS-grade methanol (30 mL) was added to the obtained powder (1 g) for an ultrasound-assisted extraction for 60 min at room temperature, with a frequency kept at 45 kHz. The solvent was renewed two times, totaling three successive extractions. The resulting extract solution was filtered, and the solvent was evaporated at 40 °C using a rotary evaporator until a dried residue was obtained. Final extracts were then stored at 4 °C until analysis by UHPLC-HRMS/MS.

### 3.4. UHPLC-ESI-Q-TOF-MS/MS

Ultra-high-performance liquid chromatography–mass spectrometry (UHPLC-MS/MS) on an Ultimate 3000-RSLC system (Thermo Scientific, Paris, France) connected to an electrospray ionization-quadrupole–time of flight (ESI-Q-TOF) instrument (Maxis II ETD, Bruker Daltonics) was used to analyze samples, calibrated using a sodium formate solution. The elution was conducted using an RSLC Polar Advantage II Acclaim column (2.2 μm, 120 Å, 2.1 × 100 mm, Thermo Scientific) with mobile phase A (milliQ water + FA 0.1%) and B (LC-MS grade CH_3_CN + FA 0.08%), at 300 μL/min, following a gradient maintaining 5% B for 2 min, 5–75% B in 8 min, then 75–95% B in 6 min, to maintaining 95% B for 2 min and then stopping the run at 22 min after returning at 5% B. The column oven was set at 40 °C. The extracts were solubilized in methanol at a concentration of 10 mg/mL and then centrifuged for 5 min at 12,000 rpm. The supernatants were collected, and 2 µL were injected. The MS detection was carried out in positive mode in the range *m*/*z* 50–1300. Analyses were performed using collision-induced dissociation in data-dependent auto-MS/MS mode. ESI source parameters were set as follows: source voltage at 3500 V, nebulizer pressure at 35 psi, drying gas flow rate at 8 L.min^−1^, and drying gas temperature at 200 °C. MS2 parameters were as follows: cycle time 3 s, an absolute threshold of 500 counts, and MS/MS speed spectra acquisition between 4 to 2 Hz. Each selected parent ion was fragmented using ramp collision energy from 30 to 50 eV.

### 3.5. Molecular Network and Peak Annotation

A molecular network was created using the online workflow (https://ccms-ucsd.github.io/GNPSDocumentation/, accessed on 1 May 2025) on the GNPS website (http://gnps.ucsd.edu, accessed on 1 May 2025). The data were filtered by removing all MS/MS fragment ions within ±17 Da of the precursor *m*/*z*. MS/MS spectra were window-filtered by choosing only the top 6 fragment ions in the +/− 50 Da window throughout the spectrum. The precursor ion mass tolerance was set to 2.0 Da, and an MS/MS fragment ion tolerance of 0.5 Da. A network (https://gnps.ucsd.edu/ProteoSAFe/status.jsp?task=5b4a98b182df4ae19f4d28f7b5f01d52, accessed on 1 May 2025) was then created where edges were filtered to have a cosine score above 0.7 and more than 6 matched peaks. Further, edges between two nodes were kept in the network if and only if each of the nodes appeared in each other’s respective top 10 most similar nodes. Finally, the maximum size of a molecular family was set to 100, and the lowest-scoring edges were removed from molecular families until the molecular family size was below this threshold. The spectra in the network were then searched against GNPS’ spectral libraries. The library spectra were filtered in the same manner as the input data. All matches kept between network spectra and library spectra were required to have a score above 0.7 and at least 6 matched peaks. The results were downloaded and exported to be visualized on Cytoscape 3.10.1 software. Compound annotation was also supported by an in-house database built from all described PPAPs [7,11]. It comprises 159 PPAP structures previously reported in *Hypericum*, including their molecular formulas and exact masses. The MS/MS fragmentation patterns of these compounds, when available in the literature or spectral libraries, were used to assist in manual dereplication. Although no experimental validation of the full database was performed, putative annotations were supported by spectral similarity, accurate mass measurements, and known occurrence of the compounds in *Hypericum* species. MS/MS spectra for each proposed PPAP are presented in the supporting information.

## 4. Conclusions

In this study, we described an approach combining automated annotation tools with targeted dereplication based on MS/MS fragmentation pathway studies to identify PPAPs in *Hypericum* species, specifically *H. patulum* and *H. hookeranium*. A molecular network provided two main clusters attributed to *O*-glycosylated flavonoids and PPAPs. While automated annotation faced challenges for PPAPs, in-house spectral data enabled the annotation of 22 peaks for both species. Key compounds such as hyperforin, hyperscabrone K, and garcinialliptone M were detected in both species, emphasizing their chemical similarity. MS/MS fragmentation pathways, particularly the successive losses of isoprenyl and isobutene units, emerged as a consistent signature for PPAP detection. The established MS/MS fingerprints may offer a practical tool for selecting extracts or fractions enriched in PPAPs for further detailed phytochemical investigations.

## Figures and Tables

**Table 1:** Annotated compounds on the molecular network of *H. patulum* and *H. hookeranium* extracts using GNPS’ spectral library. Δ (ppm) represents the difference between the experimental and theoretical mass values (in parts per million) for the detected ion.

RT (min)	Exact Mass	[M + H]^+^_the_	[M + H]^+^_exp_	Molecular Formula	Δ (ppm)	Compound Assignment	Cosine Score	Species	Reference
4.83/5.29	354.0951	355.1029	355.1048	C_16_H_18_O_9_	5.35	Chlorogenic acid	0.90	*H. patulum*, *H. hookeranium*	[17,18]
4.81	578.1424	579.1502	579.1523	C_30_H_26_O_12_	3.63	Procyanidin B2	0.89	*H. patulum*	[19]
5.22/6.17	464.0955	465.1033	465.1051	C_21_H_20_O_12_	3.87	Isoquercitrin	0.93	*H. patulum*, *H. hookeranium*	[17,18]
5.57/6.39	448.1006	449.1084	449.1090	C_21_H_20_O_11_	1.33	Quercitrin	0.92	*H. patulum*, *H. hookeranium*	[17,18]
5.47/6.36	550.0959	551.1037	551.1057	C_24_H_22_O_15_	3.63	Quercetin-3-*O*-malonylglucoside	0.92	*H. patulum*, *H. hookeranium*	[20]
5.44/6.31	434.0849	435.0927	435.0934	C_20_H_18_O_11_	1.61	Quercetin-3-*O*-arabnoside	0.93	*H. patulum*, *H. hookeranium*	[19]
5.45	578.1424	579.1502	579.1477	C_30_H_26_O_12_	4.32	Procyanidin B1	0.78	*H. hookeranium*	[19]
6.34/6.62	302.0426	303.0505	303.0522	C_15_H_10_O_7_	5.61	Quercetin	0.79	*H. patulum*, *H. hookeranium*	[17,18]

**Table 2:** Putative identification of PPAPs in *H. patulum* extract. All MS/MS fragments present experimental mass consistent with theoretical values.

RT (min)	Exact Mass	[M + H]^+^_the_	[M + H]^+^_exp_	Molecular Formula	Δ (ppm)	MS/MS Fragments	Putative Identification
13.05	500.2927	501.3005	501.2994	C_33_H_40_O_4_	2.19	433.2378, 365.1768, 309.1133, 231.0666, 189.0220	Hyperscabrone K
15.84	552.3815	553.3893	553.3916	C_35_H_52_O_5_	4.15	485.3294, 427.2862, 361.2033, 293.1403, 235.0984	Uralione A
15.99	602.3607	603.3686	603.3672	C_38_H_50_O_6_	2.28	479.2402, 445.2375, 379.1901, 311.1286, 255.0661, 177.0195	Hypercohin J
16.20	586.3658	587.3737	587.3739	C_38_H_50_O_5_	0.43	519.3101, 395.1855, 327.1231, 309.1129, 255.0655, 177.0185	Uralione C, Uralione B
16.42	566.3971	567.4050	567.4046	C_36_H_54_O_5_	0.70	499.3445, 443.2833, 375.2188, 307.1549, 217.0867	Hyphenrone E
16.46	518.3032	519.3111	519.3088	C_33_H_42_O_5_	4.26	433.2396, 377.1756, 311.1283, 255.0652, 177.0190	Sampsonione O, Hypersampsone U
17.27	502.3083	503.3161	503.3159	C_33_H_42_O_4_	0.40	447.2530, 379.1919, 311.1290, 255.0659, 177.0193	Hypersampsone T
17.56	484.3189	485.3267	485.3260	C_30_H_44_O_5_	2.09	467.3212, 399.2554, 343.1917, 277.1448, 221.0817, 163.0404	Garcinialliptone M
17.61	568.3764	569.3842	569.3846	C_35_H_52_O_6_	0.65	551.3711, 451.3711, 277.1447, 203.1810, 163.0480	Hyperforatin, 15-*epi*-hyperforatin
18.24	498.3345	499.3424	499.3404	C_31_H_46_O_5_	3.86	413.2709, 357.2064, 275.1654, 219.1019, 163.0401	Hypermongone C, Hypermongone D
18.28	536.3866	537.3944	537.3932	C_35_H_52_O_4_	2.11	481.3308, 413.2707, 277.1447, 221.0818, 165.0554	Hyperforin, Hypercohin G
18.40	468.3240	469.3318	469.3316	C_30_H_44_O_4_	0.43	413.2696, 345.2064, 277.1447, 221.0818, 165.0554	Prolifenone B
19.01	570.3709	571.3787	571.3779	C_38_H_50_O_4_	1.40	503.3243, 447.2563, 379.1930, 311.1294, 255.0662, 177.0195	Garcimultiflorone A
19.28	482.3396	483.3474	483.3480	C_31_H_46_O_4_	1.09	427.2894, 359.2233, 291.1617, 235.0978, 163.0407	Hyperibine J

**Table 3:** Putative identification of PPAPs in *H. hookeranium* extract. All MS/MS fragments present experimental mass consistent with theoretical values.

RT (min)	Exact Mass	[M + H]^+^_the_	[M + H]^+^_exp_	Molecular Formula	Δ (ppm)	MS/MS Fragments	Putative Identification
13.49	518.3032	519.3111	519.3088	C_33_H_42_O_5_	4.43	327.1225, 255.0647, 231.0637, 177.0182	Oblongifolin S
13.64	500.2927	501.3005	501.2976	C_33_H_40_O_4_	5.78	433.2335, 309.1100, 231.0638, 189.0179	Hyperscabrone K
14.39	518.3032	519.3111	519.3066	C_33_H_42_O_5_	8.67	377.1747, 311.1279, 255.0647, 177.0182	Sampsonione O isomer, Hypersampsone U isomer
16.89	518.3032	519.3111	519.3085	C_33_H_42_O_5_	5.01	377.1747, 311.1279, 255.0647, 177.0182	Sampsonione O isomer, Hypersampsone U isomer
17.55	502.3083	503.3161	503.3155	C_33_H_42_O_4_	1.19	311.1279, 255.0647, 177.0182	Oblongifolin L isomer, Oblongifolin A isomer
17.76	502.3083	503.3161	503.3155	C_33_H_42_O_4_	1.19	311.1279, 255.0647, 177.0182	Oblongifolin L isomer, Oblongifolin A isomer
17.91	484.3189	485.3267	485.3256	C_30_H_44_O_5_	2.27	343.1898, 277.1435, 221.0799, 163.0388	Garcinialliptone M
18.67	536.3866	537.3944	537.3921	C_35_H_52_O_4_	4.28	481.3294, 413.2678, 277.1435, 221.0799, 165.0540	Hyperforin, hypercohin G

## Data Availability

The data presented in this study are available on request from the corresponding author.

## Supplementary Materials

The following supporting information can be downloaded at https://www.mdpi.com/article/10.3390/molecules30122531/s1, Figure S1: MS/MS spectra (ESI, positive mode) of 3-*O*-glycosylated flavonoids annotated from GNPS database; Figure S2: MS/MS spectra (ESI, positive mode) of other phenolic compounds annotated from GNPS database; Figure S3. MS/MS spectra (ESI, positive mode) of all isopropyl-substituted PPAPs putatively identified; Figure S4: MS/MS spectra (ESI, positive mode) of all phenyl-substituted PPAPs putatively identified; Figure S5: MS/MS-ESI(+) fragmentation pathway for hyphenrone E [M + H]^+^ (567.4050 *m*/*z*) detected in *H. patulum* extract; Figure S6. Molecular Network of *H. patulum* (purple) and *H. hookeranium* (red) extracts using GNPS platform and visualized with Cytoscape 3.10.1 software; Figure S7. ^1^H and ^13^C NMR (300 and 75 MHz, respectively, CDCl3) spectra of hyperforine, used as model compound.

## References

[1] Robson N.K.B. (2016). And Then Came Molecular Phylogenetics—Reactions to a Monographic Study of Hypericum (Hypericaceae). Phytotaxa.

[2] Zhang R., Ji Y., Zhang X., Kennelly E.J., Long C. (2020). Ethnopharmacology of *Hypericum* Species in China: A Comprehensive Review on Ethnobotany, Phytochemistry and Pharmacology. J. Ethnopharmacol..

[3] Bridi H., Pustay A.P., Bordignon S.A.d.L., Picoli S.U., von Poser G.L., Ferraz A.d.B.F. (2022). Antimicrobial Activity of Dimeric Acylphloroglucinols Isolated from Southern Brazilian *Hypericum* Species against to Resistant Bacterial. Nat. Prod. Res..

[4] Ma W., Ren F., Yan X., Wang X., Wu T., Li N. (2024). Cytotoxic and Anti-Inflammatory Constituents from Roots of *Hypericum beanii* and the Antitumor Potential under the View of Cancer-Related Inflammation. Fitoterapia.

[5] Ramalhete N., Machado A.M., Serrano R., Gomes E., Mota-Filipe H., Silva O. (2016). Comparative Study on the in Vivo Antidepressant Activities of the Portuguese *Hypericum foliosum*, *Hypericum androsaemum* and *Hypericum perforatum* Medicinal Plants. Ind. Crops Prod..

[6] Rezaie A., Dorostkar K.R., Pashazadeh M., Nejad S.M. (2008). Study of Sedative and Anxiolytic Effects of Herbal Extract *Hypericum perforatum* in Comparison with Diazepam in Rats. Int. J. Infect. Dis..

[7] Bridi H., Meirelles G.d.C., von Poser G.L. (2018). Structural Diversity and Biological Activities of Phloroglucinol Derivatives from *Hypericum* Species. Phytochemistry.

[8] Cirak C., Seyis F. (2023). Phenolic Constituents of Six *Hypericum* Species from Türkiye and Their Chemotaxonomic Relevance. S. Afr. J. Bot..

[9] Manning K., Petrunak E., Lebo M., González-Sarrías A., Seeram N.P., Henry G.E. (2011). Acylphloroglucinol and Xanthones from *Hypericum ellipticum*. Phytochemistry.

[10] Seyis F., Yurteri E., Özcan A., Cirak C., Yayla F. (2022). Volatile Secondary Metabolites of *Hypericum tetrapterum* and *Hypericum bithynicum*. Biochem. Syst. Ecol..

[11] Yang X.-W., Grossman R.B., Xu G. (2018). Research Progress of Polycyclic Polyprenylated Acylphloroglucinols. Chem. Rev..

[12] Wang M., Carver J.J., Phelan V.V., Sanchez L.M., Garg N., Peng Y., Nguyen D.D., Watrous J., Kapono C.A., Luzzatto-Knaan T. (2016). Sharing and Community Curation of Mass Spectrometry Data with Global Natural Products Social Molecular Networking. Nat. Biotechnol..

[13] Morehouse N.J., Clark T.N., McMann E.J., van Santen J.A., Haeckl F.P.J., Gray C.A., Linington R.G. (2023). Annotation of Natural Product Compound Families Using Molecular Networking Topology and Structural Similarity Fingerprinting. Nat. Commun..

[14] Nothias L.-F., Nothias-Esposito M., Da Silva R., Wang M., Protsyuk I., Zhang Z., Sarvepalli A., Leyssen P., Touboul D., Costa J. (2018). Bioactivity-Based Molecular Networking for the Discovery of Drug Leads in Natural Product Bioassay-Guided Fractionation. J. Nat. Prod..

[15] Altay A., Yeniceri E., Taslimi P., Taskin-Tok T., Yilmaz M.A., Koksal E. (2022). LC-MS/MS Analysis and Diverse Biological Activities of *Hypericum scabrum* L.: In Vitro and In Silico Research. S. Afr. J. Bot..

[16] Peron G., Hošek J., Rajbhandary S., Pant D.R., Dall’Acqua S. (2019). LC-MSn and HR-MS Characterization of Secondary Metabolites from *Hypericum japonicum* Thunb. Ex Murray from Nepalese Himalayan Region and Assessment of Cytotoxic Effect and Inhibition of NF-κB and AP-1 Transcription Factors in Vitro. J. Pharm. Biomed. Anal..

[17] Alahmad A., Alghoraibi I., Zein R., Kraft S., Dräger G., Walter J.-G., Scheper T. (2022). Identification of Major Constituents of *Hypericum perforatum* L. Extracts in Syria by Development of a Rapid, Simple, and Reproducible HPLC-ESI-Q-TOF MS Analysis and Their Antioxidant Activities. ACS Omega.

[18] Tatsis E.C., Boeren S., Exarchou V., Troganis A.N., Vervoort J., Gerothanassis I.P. (2007). Identification of the Major Constituents of *Hypericum perforatum* by LC/SPE/NMR and/or LC/MS. Phytochemistry.

[19] Tocci N., Perenzoni D., Iamonico D., Fava F., Weil T., Mattivi F. (2018). Extracts from *Hypericum hircinum* Subsp. Majus Exert Antifungal Activity Against a Panel of Sensitive and Drug-Resistant Clinical Strains. Front. Pharmacol..

[20] Abdalla M.A., Famuyide I., Wooding M., McGaw L.J., Mühling K.H. (2022). Secondary Metabolite Profile and Pharmacological Opportunities of Lettuce Plants Following Selenium and Sulfur Enhancement. Pharmaceutics.

[21] Cottet K., Xu B., Coric P., Bouaziz S., Michel S., Vidal M., Lallemand M.-C., Broussy S. (2016). Guttiferone A Aggregates Modulate Silent Information Regulator 1 (SIRT1) Activity. J. Med. Chem..

[22] Henry G.E., Raithore S., Zhang Y., Jayaprakasam B., Nair M.G., Heber D., Seeram N.P. (2006). Acylphloroglucinol Derivatives from *Hypericum prolificum*. J. Nat. Prod..

[23] Mitsopoulou K.P., Vidali V.P., Maranti A., Couladouros E.A. (2015). Isolation and Structure Elucidation of Hyperibine J [Revised Structure of Adhyperfirin (7-Deprenyl-13-Methylhyperforin)]: Synthesis of Hyperibone J. Eur. J. Org. Chem..

[24] Li X., Li Y., Luo J., Zhou Z., Xue G., Kong L. (2017). New Phloroglucinol Derivatives from the Whole Plant of *Hypericum uralum*. Fitoterapia.

[25] Li H., Meng X., Zhang L., Zhang B., Liu X., Fu W., Tan H., Lao Y., Xu H. (2017). Oblongifolin C and Guttiferone K Extracted from *Garcinia yunnanensis* Fruit Synergistically Induce Apoptosis in Human Colorectal Cancer Cells in Vitro. Acta Pharmacol. Sin..

[26] Wang M., Dong Q., Wang H., He Y., Chen Y., Zhang H., Wu R., Chen X., Zhou B., He J. (2016). Oblongifolin M, an Active Compound Isolated from a Chinese Medical Herb Garcinia Oblongifolia, Potently Inhibits Enterovirus 71 Reproduction through Downregulation of ERp57. Oncotarget.

[27] Zanoli P. (2004). Role of Hyperforin in the Pharmacological Activities of St. John’s Wort. CNS Drug Rev..

[28] Koeberle A., Rossi A., Bauer J., Dehm F., Verotta L., Northoff H., Sautebin L., Werz O. (2011). Hyperforin, an Anti-Inflammatory Constituent from St. John’s Wort, Inhibits Microsomal Prostaglandin E2 Synthase-1 and Suppresses Prostaglandin E2 Formation in Vivo. Front. Pharmacol..

[29] Schempp C.M., Pelz K., Wittmer A., Schöpf E., Simon J.C. (1999). Antibacterial Activity of Hyperforin from St John’s Wort, against Multiresistant *Staphylococcus aureus* and Gram-Positive Bacteria. Lancet.

[30] Ji Y., Zhang R., Bensalel J., Morcol T., Gu R., Gallego-Delgado J., Kennelly E.J., Long C. (2024). Metabolomic and Chemometric Analyses of St. John’s Wort and Related Asian *Hypericum* Species Linked to Bioactivity. J. Ethnopharmacol..

[31] Chen Q., Di L., Zhang Y., Li N. (2020). Chemical Constituents with Cytotoxic and Anti-Inflammatory Activity in *Hypericum sampsonii* and the Antitumor Potential under the View of Cancer-Related Inflammation. J. Ethnopharmacol..

