# Supplementary material for: Targeted Dereplication of H. patulum and H. hookeranium Extracts: Establishing MS/MS Fingerprints for the Identification of Polycyclic Polyprenylated Acylphloroglucinols
Source: Molecules. 2025 Jun 10;30(12):2531. doi: 10.3390/molecules30122531 (PMC12195649; doi:10.3390/molecules30122531)
Supplement: Supplementary file 1 [file molecules-30-02531-s001.zip › molecules-3654272-supplementary.pdf]

Supporting information

Targeted-dereplication of *H. patulum* and *H. hookeranium* extracts*:* establishing MS/MS fingerprints for the identification of polycyclic polyprenylated acylphloroglucinols

Annabelle Dugay [1+], Florence Souquet [1+], David Hozain [1], Gilles Alex Pakora [1], Didier Buisson [2], Séverine Amand [2], Marie-Christine Lallemand [1#] and Raimundo Gonçalves de Oliveira Junior [1#*]

[1] *Cibles Thérapeutiques et Conception de Médicaments* (CiTCoM UMR CNRS 8038), Faculty of Pharmacie, Université Paris Cité, Paris, France.
[2] *Molécules de Communication et Adaptation des Micro-organismes* (MCAM UMR CNRS 7245), Muséum National d'Histoire Naturelle (MNHN), Paris, France.
[+] Both authors equally contributed as first authors.
[#] Both authors equally contributed to the supervision of this work.
* Correspondence: raimundo.goncalves-de-oliveira-junior@u-paris.fr

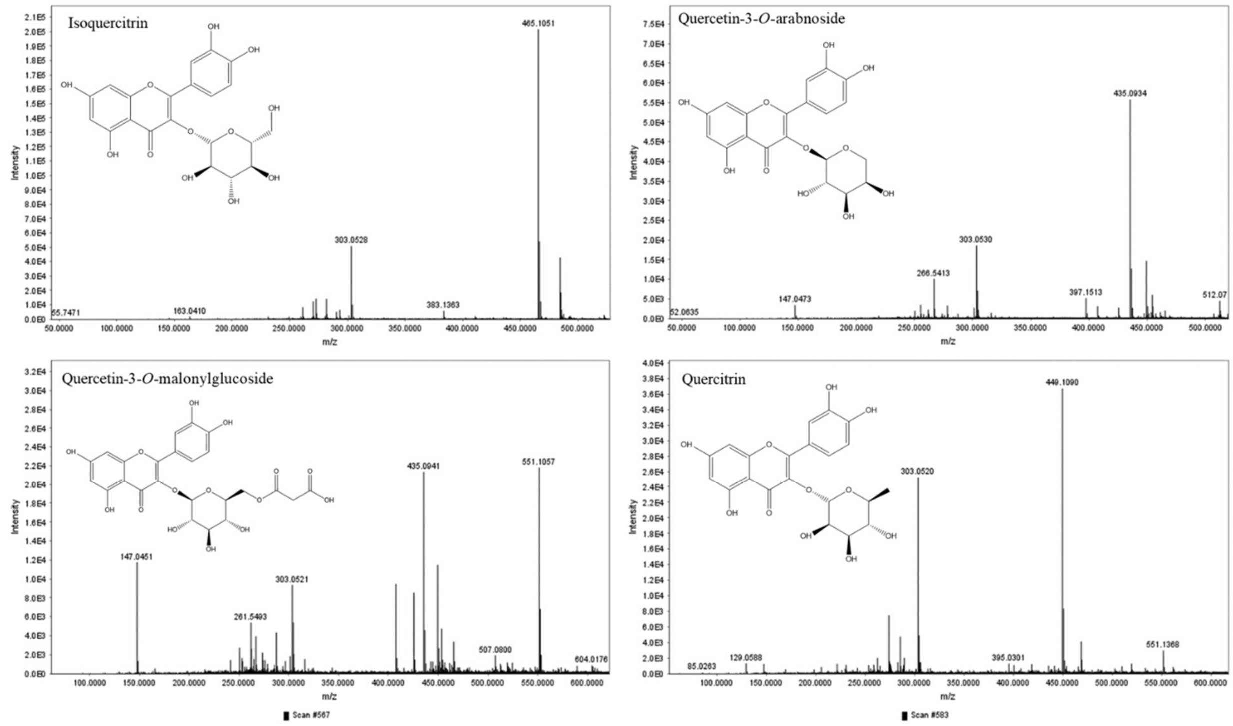

Figure S1. MS/MS spectra (ESI, positive mode) of 3-*O*-glycosylated flavonoids annotated from GNPS database.

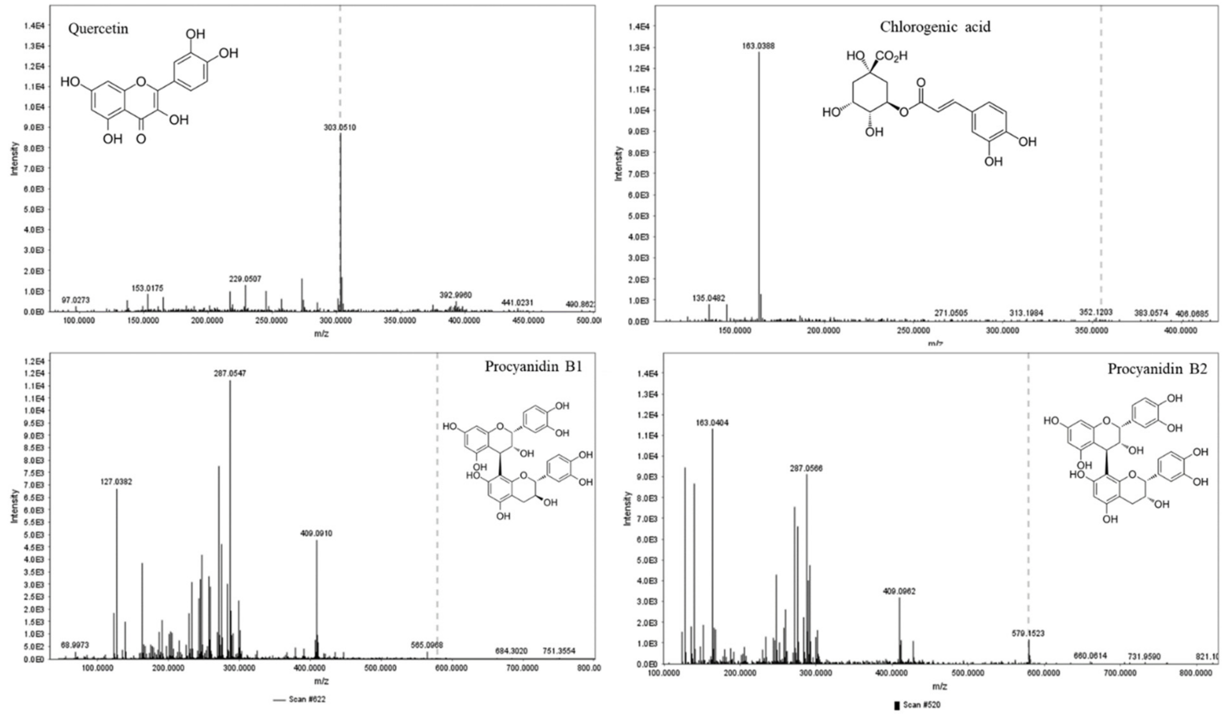

Figure S2. MS/MS spectra (ESI, positive mode) of other phenolic compounds annotated from GNPS database.

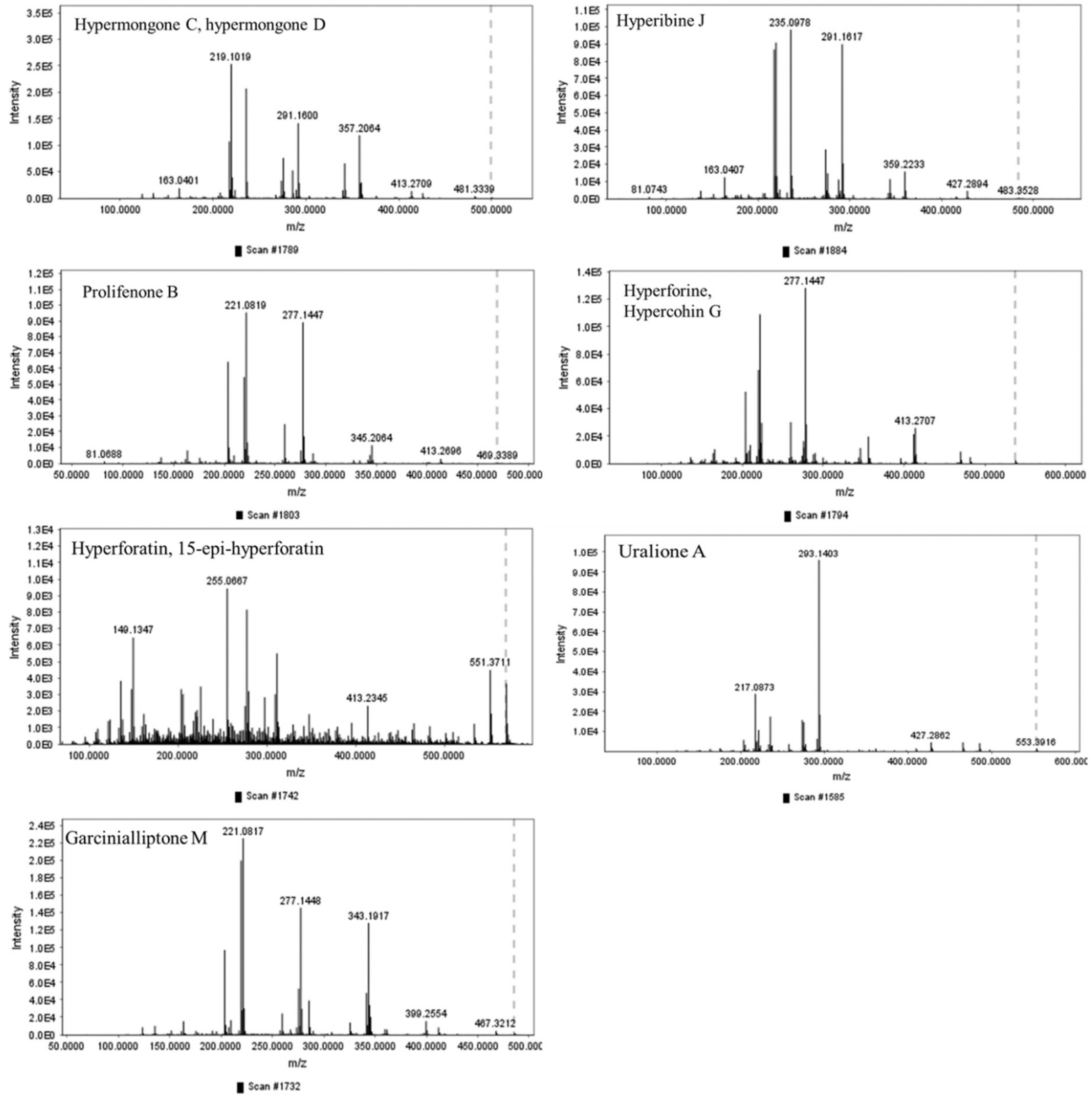

Figure S3. MS/MS spectra (ESI, positive mode) of all isopropyl-substituted PPAPs putatively identified.

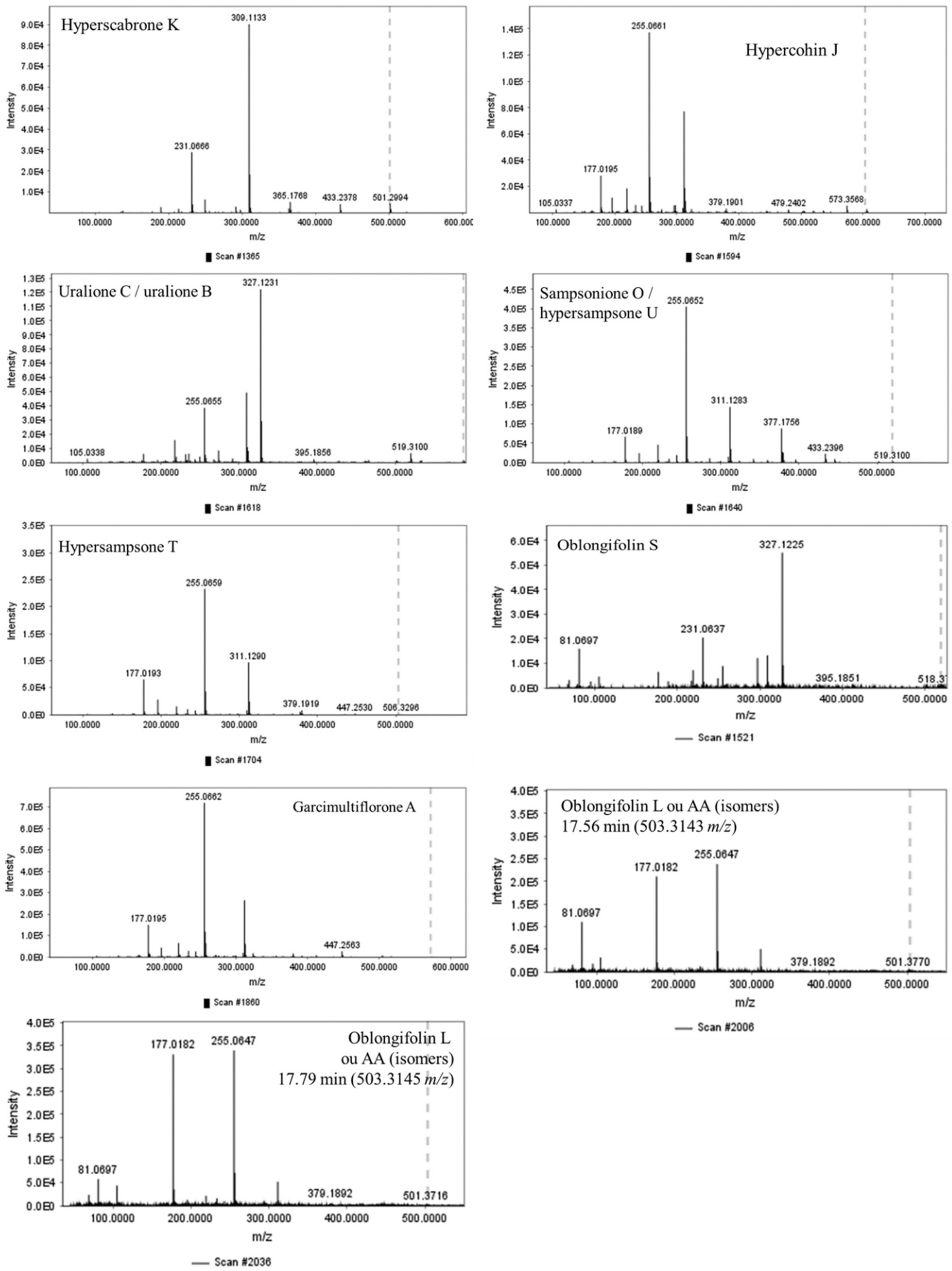

Figure S4. MS/MS spectra (ESI, positive mode) of all phenyl-substituted PPAPs putatively identified.

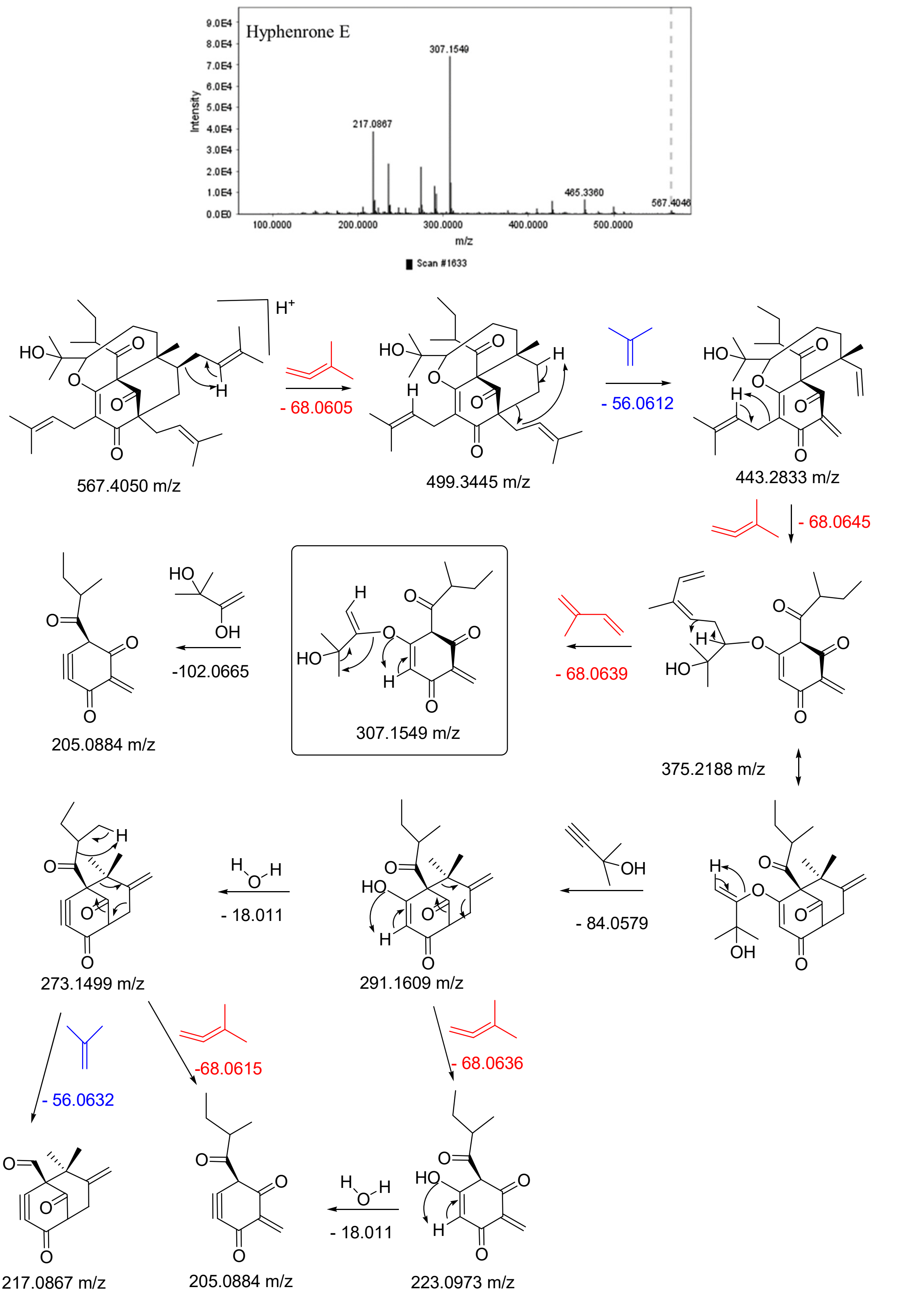

Figure S5. MS/MS-ESI(+) fragmentation pathway for hyphenrone E $[M+H]^+$ (567.4050 *m/z*) detected in *H. patulum* extract.

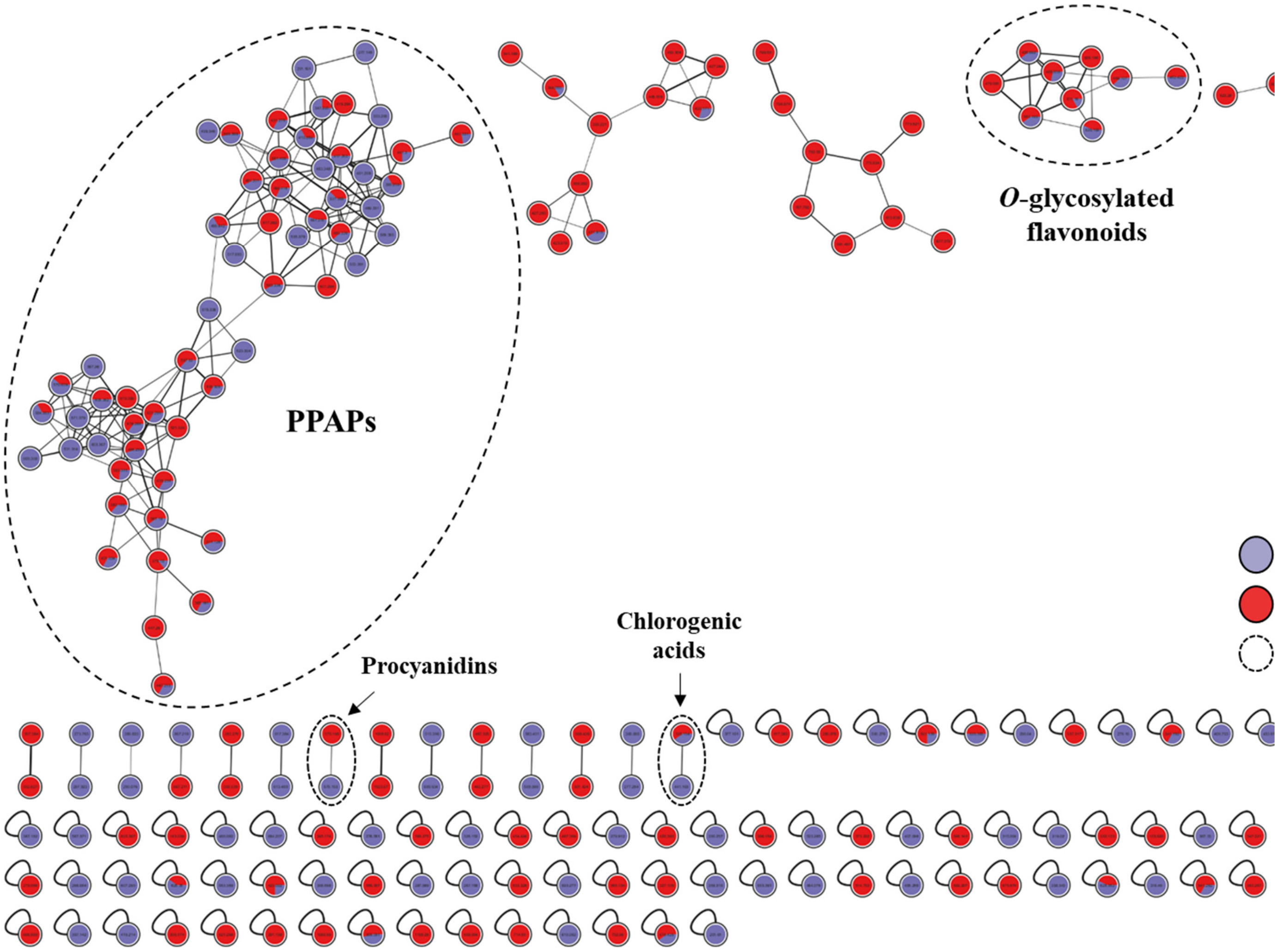

Figure S6. Molecular Network of *H. patulum* (purple) and *H. hookeranium* (red) extracts using GNPS platform and visualized with Cytoscape 3.10.1 software.

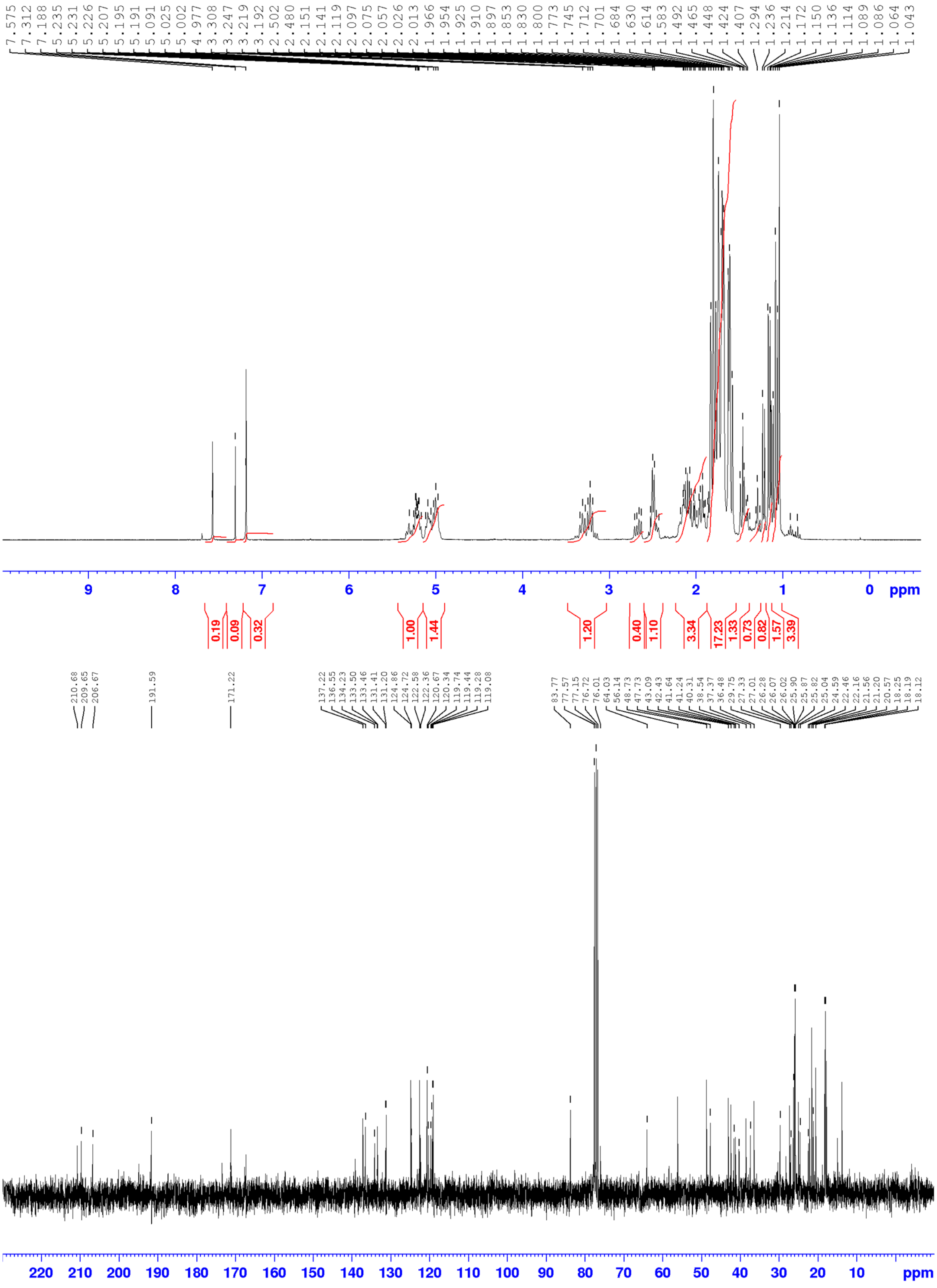

Figure S7. ^{1}H and ^{13}C NMR (300 and 75 MHz, respectively, CDCl3) spectra of hyperforine, used as model compound.
